# Supplementary material for: Evaluating Antivenom Efficacy against Echis carinatus Venoms—Screening for In Vitro Alternatives
Source: Toxins (Basel). 2022 Jul 13;14(7):481. doi: 10.3390/toxins14070481 (PMC9322380; doi:10.3390/toxins14070481)
Supplement: Supplementary file 1 [file toxins-14-00481-s001.zip › toxins-1764676-supplementary.pdf]

toxins

Supplementary materials

Evaluating Antivenom Efficacy against *Echis carinatus* Venoms—Screening for In Vitro Alternatives

Siddharth Bhatia †, Avni Blotra † and Karthikeyan Vasudevan *

CSIR-Centre for Cellular and Molecular Biology, Laboratory for the Conservation of Endangered Species, Hyderabad, India; siddharth.bhatia17@hotmail.com (S.B.); avniblotra76@gmail.com (A.B.)
* Correspondence: karthik@ccmb.res.in
† These authors contributed equally to this work.

Table S1. Binding parameters for hyperbolic curve fitted AUC vs Antivenom-venom amounts plot (Figure 2) are listed above where AUC_{max} is the asymptotic maxima for AUC_{Z1} and EC50 is antivenom-venom ratio at which AUCZ1 was half the AUC_{max}.

	ECVTN	ECVGO	ECVRAJ
**Best-fit values**			
AUC_{max}	600.1	262.4	467.4
EC50	118.2	92.73	177.4
**95% CI (profile likelihood)**			
AUC_{max}	463.0 to 897.0	187.1 to 456.2	399.1 to 569.5
EC50	70.94 to 229.4	45.19 to 238.8	136.5 to 239.9
**Goodness of Fit**			
Degrees of Freedom	22	22	14
R squared	0.9562	0.87	0.9953

Table S2. Number of $F(ab)_2$ molecules per venom toxin is estimated using the formula described in methodology section 2.2. Average molecular weight of toxin for each venom group was estimated by densitometry analysis of SDS-PAGE bands reported in (Bhatia and Vasudevan, 2020). Molecular weight of $F(ab)_2$ moleculeswere taken as 110 kDa.

Venom	Avg molecular weight (in kDa)	Venom amount (in ng)	Venom amount in nmols (Ag)	Max antivenom bound (in ng)	Amount of antibody in nmols (Ab)	Number of antibodies per venom toxins (Ab/Ag)
ECVTN	45.16	10000	0.22	95700	0.87	4.0
ECVGO	55.19	10000	0.18	13400	0.122	0.67
ECVRAJ	33.66	10000	0.30	62300	0.566	1.89

Table S3. Clotting times (CT) for different amounts of venoms. Clotting times were estimated from the plasma of two healthy donors and readings were taken in triplicate for each plasma.

	Venom amount in μg				
	**10**	**5**	**2.5**	**1.3**	**0.6**
ECVRAJ	26.9	37.3	62.7	90.4	165
	26.4	36.5	57.2	92.8	165.3
	25.5	35.5	60.5	104.1	221.8
	29.4	44	69.5	114.6	227.3
	30	43.4	72.4	109.5	239.2
	30.8	47	66	123.4	347.5
ECVTN	20	28.8	41.6	66	100
	23.5	29	45	67	121

	21.5	30	43.7	67	120
	22.5	29.7	44.3	64.9	104.7
	22.6	29.7	45.2	70.6	112.5
	21.3	30.1	47.7	72	120.8
ECVGO	18.6	20.5	28.2	38.3	60.4
	16.6	21	28.2	42.8	63.3
	16.4	22.2	27.2	39.9	57.4
	15.6	20.7	28.8	42.5	62.8
	15.1	19.8	27.2	40.8	57.5
	16.1	19.9	27.1	37.1	55.4

Table S4. Fold change in the clotting times (CT) for different antivenom-venom ratios are represented in the table above. It is calculated by dividing CT of antivenom-venom dilution with CT of challenge dose of venom (2*MCD). The readings were taking in triplicates for the plasma from 2 volunteers.

	Antivenom-venom Ratios				
	**100:1**	**50:1**	**25:1**	**12.5:1**	**6.25:1**
ECVRAJ		8.01	2.14	1.22	1.01
		8.3	2.03	1.24	1.06
	no clot seen	7.76	1.97	1.23	
		8.82	2.08	1.25	1.04
		8.82	2.12	1.34	1.02
ECVTN	4.91	2.17	1.38	1.25	1.14
	5.04	2.15	1.35	1.21	1.13
	5.25	2.13	1.32	1.26	1.2
	5.82	2.43	1.41	1.14	1.04
	5.23	2.41	1.52	1.14	1.07
	5.74	2.44	1.41	1.17	1.08
ECVGO	9.08	4.84	1.6	1.37	1.22
	8.22	4.4	1.41	1.21	1.07
	7.79	3.99	1.35	1.29	1.07
	8.97	3.74	1.47	1.2	1.15
	8.35	3.53	1.42	1.08	0.98
	8.91	3.7	1.45	1.15	1.09

Table S5. Percentage decrease in proteolytic activity referred as %R were estimated using formula described in methodology section 2.3.2. The table represents %R (n=3 for ECVTN and ECVGO; n=2 for ECVRAJ) for different antivenom-venom ratios.

**Antivenom-venom ratio**	**ECVTN**			**ECVGO**			ECVRAJ	
168:1	72.80	75.54	74.60	72.34	64.33	70.17	23.93	38.08
84:1	69.65	65.00	63.99	65.15	40.01	58.97	36.35	25.74
42:1	46.64	43.26	46.47	50.38	11.28	28.61	14.75	23.03
21:1	41.11	36.65	28.12	32.17	14.00	24.59	22.12	22.12
11.5:1	25.31	26.54	15.85	19.01	3.29	6.35	-1.46	8.17
5.75:1	18.87	19.96	9.91	16.52	12.06	5.95	5.31	0.49

Table S6. Percentage decrease in PLA_2 activity (%R) calculated by the formula described in methodology section 2.3.2. at different antivenom-venom ratio is represented for each venom group (n=3 for ECVTN and ECVGO; n=2 for ECVRAJ).

Antivenom-venom ratio	ECVTN			ECVGO			ECVRAJ	
100:1	89.79	94.40	96.55	95.72	96.42	96.86	15.08	28.51
50:1	93.65	95.38	96.43	77.05	74.28	73.91	22.25	47.43
25:1	89.13	90.76	92.37	55.33	60.60	66.12	13.66	25.54
12.5:1	62.82	69.94	69.14	22.37	28.69	32.41	13.62	25.35
6.25:1	52.90	52.39	55.53	28.78	18.18	18.25	24.57	21.40

Table S7. Parameters for sigmoidal curve fitted to the %R vs Log(antivenom-venom ratio) plot are represented in the table. $\%R_{max}$ is maximum reduction in the activity and IC50 is antivenom-venom ratio at which $\%R_{max}$ is half.

List of assays	Venom	Curve fitting parameters		
		$\%R_{max}$	IC50	R squared
PLA_2 Assay	ECVTN	94.42	14.39	0.983
	ECVGO	105.70	27.38	0.965
	ECVRAJ	27.32	Unstable	0.153
Proteolytic assay	ECVTN	86.97	42.35	0.964
	ECVGO	81.88	64.79	0.837
	ECVRAJ	32.51	18.76	0.764
Coagulation assay	ECVTN	102.60	43.90	0.991
	ECVGO	100.10	28.86	0.994
	ECVRAJ	101.50	24.61	0.995
